# Cardiovascular Outcomes among Combustible-Tobacco and Electronic Nicotine Delivery System (ENDS) Users in Waves 1 through 5 of the Population Assessment of Tobacco and Health (PATH) Study, 2013–2019

**DOI:** 10.3390/ijerph19074137

**Published:** 2022-03-31

**Authors:** Martin C. Mahoney, Cheryl Rivard, Heather L. Kimmel, Hoda T. Hammad, Eva Sharma, Michael J. Halenar, Jim Sargent, K. Michael Cummings, Ray Niaura, Maciej L. Goniewicz, Maansi Bansal-Travers, Dorothy Hatsukami, Diann Gaalema, Geoffrey Fong, Shannon Gravely, Carol H. Christensen, Ryan Haskins, Marushka L. Silveira, Carlos Blanco, Wilson Compton, Cassandra A. Stanton, Andrew Hyland

**Affiliations:** 1 Roswell Park Comprehensive Cancer Center, Buffalo, NY 14263, USA; martin.mahoney@roswellpark.org (M.C.M.); maciej.goniewicz@roswellpark.org (M.L.G.); maansi.travers@roswellpark.org (M.B.-T.); andrew.hyland@roswellpark.org (A.H.); 2 National Institute on Drug Abuse, National Institutes of Health, Bethesda, MS 20892, USA; heather.kimmel@nih.gov (H.L.K.); marushka.silveira@nih.gov (M.L.S.); carlos.blanco2@nih.gov (C.B.); wcompton@nida.nih.gov (W.C.); 3 Center for Tobacco Products, U.S. Food and Drug Administration, Silver Spring, MD 20993, USA; hoda.hammad@fda.hhs.gov (H.T.H.); carol.christensen@fda.hhs.gov (C.H.C.); ryan.haskins@fda.hhs.gov (R.H.); 4 Westat, Rockville, MD 20850, USA; evasharma@westat.com (E.S.); michaelhalenar@westat.com (M.J.H.); cassandrastanton@westat.com (C.A.S.); 5 The C. Everette Koop Institute at Dartmouth, Geisel School of Medicine at Dartmouth, Lebanon, NH 03756, USA; james.d.sargent@dartmouth.edu; 6 Medical University of South Carolina, Charleston, SC 29425, USA; cummingk@musc.edu; 7 NYU School of Global Public Health, New York, NY 10003, USA; rn54@nyu.edu; 8 University of Minnesota, Minneapolis, MN 55414, USA; hatsu001@umn.edu; 9 University of Vermont, Burlington, VT 05404, USA; diann.gaalema@uvm.edu; 10 University of Waterloo, Waterloo, ON N2L 3G1, Canada; geoffrey.fong@uwaterloo.ca (G.F.); shannon.gravely@uwaterloo.ca (S.G.); 11 Kelly Government Solutions, Troy, MI 48084, USA

**Keywords:** tobacco use, cardiovascular disease, health survey, electronic nicotine delivery systems (ENDS), electronic cigarette

## Abstract

Background: Prior studies have not clearly established risk of cardiovascular disease (CVD) among smokers who switch to exclusive use of electronic nicotine delivery systems (ENDS). We compared cardiovascular disease incidence in combustible-tobacco users, those who transitioned to ENDS use, and those who quit tobacco with never tobacco users. Methods: This prospective cohort study analyzes five waves of Population Assessment of Tobacco and Health (PATH) Study data, Wave 1 (2013–2014) through Wave 5 (2018–2019). Cardiovascular disease (CVD) incidence was captured over three intervals (Waves 1 to 3, Waves 2 to 4, and Waves 3 to 5). Participants were adults (40+ years old) without a history of CVD for the first two waves of any interval. Change in tobacco use status, from exclusive past 30 day use of any combustible-tobacco product to either exclusive past 30 day ENDS use, dual past 30 day use of ENDS and combustible-tobacco, or no past 30 day use of any tobacco, between the first two waves of an interval was used to predict onset of CVD between the second and third waves in the interval. CVD incidence was defined as a new self-report of being told by a health professional that they had congestive heart failure, stroke, or a myocardial infarction. Generalized estimating equation (GEE) analyses combined 10,548 observations across intervals from 7820 eligible respondents. Results: Overall, there were 191 observations of CVD among 10,548 total observations (1.7%, standard error (SE) = 0.2), with 40 among 3014 never users of tobacco (1.5%, SE = 0.3). In multivariable models, CVD incidence was not significantly different for any tobacco user groups compared to never users. There were 126 observations of CVD among 6263 continuing exclusive combustible-tobacco users (adjusted odds ratio [AOR] = 1.44; 95% confidence interval (CI) 0.87–2.39), 15 observations of CVD among 565 who transitioned to dual use (AOR = 1.85; 0.78–4.37), and 10 observations of CVD among 654 who quit using tobacco (AOR = 1.18; 0.33–4.26). There were no observations of CVD among 53 who transitioned to exclusive ENDS use. Conclusions: This study found no difference in CVD incidence by tobacco status over three 3 year intervals, even for tobacco quitters. It is possible that additional waves of PATH Study data, combined with information from other large longitudinal cohorts with careful tracking of ENDS use patterns may help to further clarify this relationship.

## 1. Introduction

Since the 1964 Advisory Report to the Surgeon General on smoking and health, documenting the harms caused by smoking and other tobacco products has been a recurring theme of subsequent reports issued by the Surgeon General [1,2]. The 2014 Surgeon General’s report stated that cigarette smokers have a 2–4-fold higher risk of cardiovascular diseases (CVDs) (e.g., coronary heart disease, myocardial infarction (MI), hypertension, congestive health failure (CHF) and stroke) compared to non-smokers [1,2,3]. Moreover, ischemic events within the heart (e.g., MI) and brain (e.g., stroke) together accounted for ~28% of all deaths in the United States in 2016 and 2017 [4]. In addition to fatal and non-fatal acute cardiovascular events, smoking contributes to accelerated rates of atherosclerosis and sudden death [2]. Cessation of smoking reduces the risk of coronary events fairly rapidly, so that within three years of quitting, the average risk level is similar to that of someone who never smoked [5].

The mechanisms underlying increased risks of cardiovascular events, which are best characterized among cigarette smokers, but extend to users of other combusted tobacco products, are multifactorial and include exposures to harmful constituents of tobacco smoke and interactions with various physiologic processes. Tobacco smoke contains oxidizing chemicals, nicotine, carbon monoxide (CO), volatile organic compounds, particulates and heavy metals. The oxidizing compounds contribute to lipid formation, endovascular deposition and oxidative stress within blood vessels. Exposure to nicotine results in hemodynamic changes including increased blood pressure and heart rate, resulting in increased cardiac demand, while the vasoconstrictive effects of nicotine simultaneously decrease blood flow, resulting in reduced oxygen supply [1,2,6]. Nicotine also results in arrhythmogenesis and an increased risk of a fatal cardiac event [2,6]. CO reduces oxygen delivery such that increased oxygen demand is met with decreased availability [1,2]. Moreover, tobacco smoking contributes to endothelial dysfunction, hypercoagulable state/thrombosis, inflammation, insulin resistance (thereby increasing risk of diabetes in smokers), hyperlipidemia (smoking decreases high-density lipoprotein [HDL] levels and oxidizes low-density lipoprotein (LDL), leading to endovascular deposition and increased inflammation and atherosclerosis, including within coronary and cerebral vessels) [1,2,6].

Because electronic nicotine delivery systems (ENDS) are relatively new products in the marketplace, there are few longitudinal studies that have been able to explore the association between ENDS use and cardiovascular disease risk. Most of the epidemiologic studies on ENDS and CVD risks are based on cross-sectional designs using prevalence outcomes [7,8,9]. Further complicating matters is the observation that most ENDS users are former smokers, so adjusting for one’s prior smoking history is challenging.

Since many of the harms of smoking are related to the direct and indirect effects of combustion, some evidence suggests that ENDS do not appear to have major short-term health effects [10], which offers support for a “harm-reduction” strategy of switching from combusted tobacco to ENDS [7,8]. However, laboratory studies point to a potential increase in oxidative stress and changes in heart rate variability resulting from ENDS use [9,11], both of which are associated with increased cardiovascular risk [9,12]. While some of this cardiovascular risk has been attributed to the physiologic effects of nicotine, the explanation for increased oxidative stress is unclear but could be related to lipid peroxidation as evidenced by decreases in nitric oxide and increases in nicotinamide adenine dinucleotide phosphate (NADPH) oxidase and 8-iso-prostaglandin F2α noted among users of ENDS [9,11].

Since existing studies have not clearly established whether the risk of CVD changes when smokers switch to exclusive use of ENDS, we seek to address this gap using longitudinal Population Assessment of Tobacco and Health (PATH) Study data from Waves 1 to 5 (2013/14–2018/19) to compare CVD incidence among adults (age 40+ years who were either exclusive combustible-tobacco users or never users of tobacco at baseline) grouped into: (1) exclusive combustible-tobacco (including cigarettes, traditional cigars, cigarillos, filtered cigars, pipe tobacco, and hookah) users who remain exclusive combustible-tobacco users, (2) exclusive combustible-tobacco users who transitioned to exclusive ENDS use, (3) exclusive combustible-tobacco users who transitioned to dual use of ENDS and combustible-tobacco, (4) exclusive combustible-tobacco users who quit using tobacco, and (5) never users of tobacco.

## 2. Materials and Methods

The PATH Study is an ongoing, nationally representative, longitudinal cohort study of adults and youth in the United States (U.S.). The study uses audio computer-assisted self-interviews (ACASI), available in English and Spanish, to collect information on tobacco use patterns and associated health behaviors. The PATH Study recruitment for the Wave 1 Cohort employed a stratified address-based, area-probability sampling design that oversampled adult tobacco users, young adults (18 to 24 years), and African American adults. An in-person screener was used at Wave 1 (W1) to randomly select youths and adults from households for participation in the study. The total unweighted cumulative attrition rate among the W1 sample was 16% at Wave 2 (W2), 21% at Wave 3 (W3), 27% at Wave 4 (W4), and 30% at Wave 5 (W5). Differences in the number of completed interviews between Wave 1 and subsequent waves reflect respondent attrition (e.g., non-response and mortality). An analysis of non-response bias from attrition from W1 to W4 of the PATH Study (available in the PATH Study Restricted Use Files User Guide [12]) concluded “little if any non-response bias” among adults.

Full-sample and replicate weights were created to adjust for the complex sample design (e.g., oversampling of specified groups) and non-response. Weighted estimates represent the resident population of the U.S. who were in the civilian, non-institutionalized population (CNP) at W1 and W5. All-wave weights were assigned to W5 respondents in the W1 cohort, who also participated in W2, W3, and W4. Further details regarding the PATH Study design and methods for the W1 cohort are published elsewhere [13,14,15]. The analyses presented here used the Restricted Use Files (RUF). Missing data on age were imputed as described in the PATH Study Restricted Use Files User Guide, and details on interview procedures, questionnaires, sampling, weighting, response rates, and accessing the data are described in the PATH Study Restricted Use Files User Guide. The study was conducted by Westat and approved by the Westat Institutional Review Board. All respondents ages 18 and older provided informed consent.

### 2.1. Measures

#### 2.1.1. Tobacco Use

At each wave, respondents were asked about ever, past 12 month and past 30 day (P30D) tobacco use behaviors for combustible-tobacco (cigarettes, traditional cigars, cigarillos, filtered cigars, pipe tobacco, and hookah), ENDS, as well as other non-combustible tobacco (smokeless tobacco, snus pouches, and dissolvable tobacco). At W1, ENDS were described as ‘e-cigarettes that look like regular cigarettes, but are battery-powered and produce vapor instead of smoke’. At Waves 2 through 5, ENDS were described as ‘electronic nicotine products such as e-cigarettes, e-cigars, e-pipes, e-hookahs, and personal vaporizers, as well as vape pens and hookah pens that are battery-powered, use nicotine fluid rather than tobacco leaves, and produce vapor instead of smoke’. Five groups were considered based upon self-reported tobacco use: (1) continuing exclusive combustible-tobacco users (exclusive P30D use of any combustible-tobacco product (and no non-combustible-tobacco products) at each wave in an interval (n = 6263 observations); (2) exclusive combustible-tobacco users who transition to exclusive ENDS use (and no other tobacco products, n = 53 observations); (3) exclusive combustible-tobacco users who transition to dual P30D use of ENDS and combustible-tobacco (and no other tobacco products, n = 565 observations); (4) exclusive combustible-tobacco users who quit using tobacco (no P30D use of any tobacco, n = 654 observations); and (5) never users of tobacco who remain P30D non-users of tobacco (n = 3014 observations).

#### 2.1.2. Cardiovascular Risk Factors

In addition to smoking, other established risk factors for CVD include hypertension, elevated cholesterol, diabetes, obesity and family history of CVD [2,3,16,17]. In the PATH Study, adult participants were asked: ‘Has a doctor or other health professional ever told you that you had any of the following conditions?’ Responses included high blood pressure, high cholesterol and diabetes. Participants were also asked, ‘Were any of your close biological or blood relatives ever told by a health professional that they had a heart attack or needed bypass surgery?’ If yes, ‘Were they told they had a heart attack or needed bypass surgery before the age of 50?’ Finally, a body mass index (BMI) was calculated for each participant based on their self-reported height and weight. We limited respondents to those age 40+ years given the very low prevalence of CVD below this age. CVD risk factors included in our analyses are: sex (male), cigarette pack-years, family history of premature heart disease (before age 50), elevated body mass index (BMI) (≥35), and a report of ever having been diagnosed with high blood pressure, high cholesterol, and/or diabetes. These data were collected at each survey wave and used as adjustment variables in regression analysis.

#### 2.1.3. Cardiovascular Outcome Measures

CVD was measured at each wave with a series of questions in which respondents were asked, ‘Has a doctor or other health professional ever told you that you had any of the following conditions?’ Responses included congestive heart failure, stroke, heart attack (also called myocardial infarction (MI)) or needed bypass surgery, some other heart condition, none of the above (yes, no). If respondents reported CHF, stroke, or heart attack at either of the first two waves of an interval, then they were excluded from the analysis. Among the respondents who were free of CVD at the first two waves of an interval, incident cardiovascular conditions were determined at the third wave by asking participants: ‘In the past 12 months, has a doctor or other health professional told you that you had any of the following conditions?’ with the same response options. Participants who reported that they had been told they had CHF, stroke, or heart attack at a subsequent wave were classified as having an incident CVD. Our previous analyses of self-reported CVD among adults age 40 years and older using the PATH Study data established the concurrent validity and reliability of measures of CVD [18].

### 2.2. Analysis Plan

**Figure 1:**
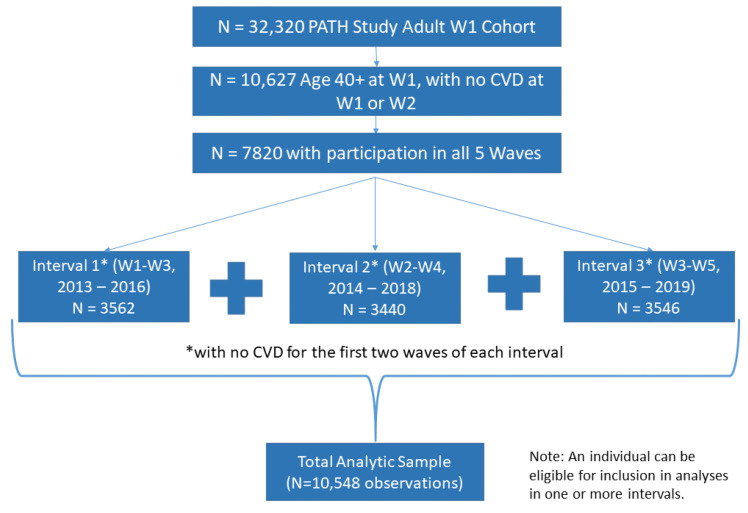
Derivation of Analytic Sample.

This study analyzes five waves of PATH Study data, beginning with W1 (2013–2014) through W5 (2018–2019) by considering CVD incidence across three wave intervals: W1 to W3, W2 to W4, and W3 to W5 in a single analysis. The analytic sample was restricted to adults 40 and older with no history of a cardiac condition at either of the first two waves of an interval and who completed all five waves of the PATH Study (n = 7820. For Interval 1 (W1–W3, n = 3562), W1 was the baseline; for Interval 2 (W2–W4, n = 3440, W2 was the baseline; and for Interval 3 (W3–W5, n = 3546), W3 was the baseline. This approach allowed us to explore number of observations instead of number of participants, resulting in the final sample of 10,548 observations (see Figure 1). Generalized estimating equations (GEE) regression analysis was performed adjusting for CVD risk factors to account for multiple observations for the same individual. This analysis used W5 all-waves weights to obtain statistically valid estimates from longitudinal analyses which examine the PATH Study W1 cohort data across Waves 1 through 5, and variances were estimated using the balanced repeated replication method [19], with Fay’s adjustment set to 0.3 [20].

Change in tobacco use category between the first two waves of a given interval was used to predict onset of CVD between the second and third waves within each interval.

## 3. Results

### 3.1. Sample Description

Table 1 presents demographic and cardiovascular risk factors among adults age 40+ years in W1 of the PATH Study by tobacco use category. Among continuing exclusive combustible-tobacco users, 55.3% were male, compared to 47.4% of those who transitioned to exclusive ENDS use, 44.2% of those who transitioned to dual use, 61.7% of those who quit using tobacco, and 32.8% of never users of tobacco (*p* < 0.001). Of the continuing exclusive combustible-tobacco users, 47.1% were age 55 or over, compared to 43.0% of those who transition to exclusive ENDS use, 33.5% of those who transition to dual use, 50.6% of those who quit using tobacco, and 54.8% of never users of tobacco (*p* < 0.001). The average number of cigarette pack-years among continuing exclusive combustible-tobacco users was 25.1 years, compared to 16.1 years among those transitioning to exclusive ENDS use, 28.0 years among those transitioning to dual use, and 11.2 years among those who quit using tobacco (*p* < 0.001). Among continuing exclusive combustible-tobacco users, 11.3% had a BMI ≥ 35, compared to 13.5% of those transitioning to exclusive ENDS use, 13.7% of those transitioning to dual use, 12.4% of those who quit using tobacco, and 14.5% of never users of tobacco (*p* = 0.01).

### 3.2. Cardiovascular Disease Incidence

Table 2 presents the results of the adjusted GEE regression models. CVD incidence was experienced by 2.1% of continuing exclusive combustible-tobacco users, 0.0% of exclusive combustible-tobacco users who transitioned to exclusive ENDS use, 2.5% of exclusive combustible-tobacco users who transitioned to dual use, 1.6% of exclusive combustible-tobacco users who quit using tobacco and 1.5% of never tobacco users. Since there was no incident CVD among exclusive combustible-tobacco users who transitioned to exclusive ENDS use, that group was not able to be analyzed further. Compared to never users of tobacco, no statistical differences in CVD incidence were observed for continuing exclusive combustible-tobacco users (adjusted odds ratio [AOR] = 1.44; 95% confidence interval (CI) 0.87–2.39), for those who transitioned to dual use (AOR = 1.85; 0.78–4.37), or for those who quit using tobacco (AOR = 1.18; 0.33–4.26). A sensitivity analysis examined frequencies for CVD incidence by cigarette pack-year groupings and found that CVD incidence was higher with more pack-years (results not shown).

## 4. Discussion

Previous research has generally reported a 2–4-fold higher risk for CVD among smokers compared to non-smokers [1,2,3]. A recent study of over 350,000 participants age 35 to 80 in the US, followed for over 10 years, reported a hazard ratio of 1.44 (95% CI 1.38–1.51) for cardiovascular disease among current and former cigarette smokers compared to never combustible-tobacco users [21]. The present study identified a non-statistically significant increased risk for CVD among continuing exclusive combustible-tobacco users compared to never tobacco users (AOR = 1.44; 95% CI 0.87–2.39).

We did not find sufficient evidence to suggest that transitioning to exclusive use of ENDS significantly changes the odds of CVD incidence, although this analysis was based on a small sample size and a relatively limited interval of follow up. We observed no incident CVD among 53 observations of exclusive combustible-tobacco users at a baseline year who transitioned to exclusive ENDS use. This limited number of adults who transitioned from combustible-tobacco to exclusive use of ENDS limits our ability to estimate health effects. Future analyses with additional waves of PATH Study data will increase the statistical power for this comparison. Alternatively, population-based studies may need to oversample exclusive ENDS users to be able to estimate risk accurately. Finally, our overall findings are consistent with a rapid change in risk of cardiovascular events (e.g., after 3 years of smoking cessation) [5].

Recent publications based on cross-sectional W1 PATH Study data reported lower levels of selected cardiovascular biomarkers (e.g., hs CRP, IL-6, sICAM, fibrinogen, and urinary 8-isoprostane) in exclusive ENDS users compared to exclusive smokers [22] and a greater concentration of 8-isoprostane among dual users of cigarettes and e-cigarettes compared to smokers [23]. These biomarkers of inflammation and oxidative stress are associated with smoking-induced CVD, and related biomarkers have been studied as predictive factors for cardiovascular events. In contrast to PATH Study data, others have reported increases in markers of oxidative stress and reduced heart rate variability from ENDS use after acute exposures [9,13].

The obvious limitations of this analysis are its relatively modest sample for several tobacco user groups (especially exclusive ENDS users), relatively young ages of adult smokers who transition to ENDS, and limited duration of follow up. Illustrative of this limitation is an example from the Framingham community study, which, based on the first 4 years of follow up from 1948 to 1950, was unable to show a significant association between smoking and heart disease [24]. It took many more years of observation to reliably establish the association between cigarette smoking and CVD risk [3]. With our study design, we can only identify new cases of CVD that occur within one survey wave of when tobacco use status changes were assessed. Nonetheless, with longer duration of follow up of the PATH Study cohort, future analyses will be able to more reliably assess the association between different patterns of tobacco use and CVD risk as well as the risk of other diseases. Another potential limitation to note is that these findings are based on participants’ self-reported CVD and tobacco use. However, our previous study of CVD among adults age 40 years and older using the PATH Study data established the concurrent validity and reliability of these self-reported measures of CVD [18]. Potential additional analyses might link a measure of biochemical verification of self-reported tobacco use. A final limitation that warrants mention is the use of tobacco products in the past 30 days as the primary measure of tobacco use. There is a wide range of use of tobacco that could classify an individual as a tobacco user, and the PATH Study includes several measures of frequency of use for each tobacco product. However, we determined that past 30 day use was the best measure to use for this analysis in order to include as many observations as possible.

Strengths of this report include the PATH Study data and the use of robust statistical models in the analyses. The use of GEE analyses with “stacked data” over several intervals allowed for the inclusion of observations from three time intervals in a single analysis while statistically controlling for interdependence among observations contributed by the same individual [25,26]. ENDS users tend to be younger than non-users and age adjustment might not eliminate this effect if it is a systematic difference among groups, and data on exposures to marijuana and second hand smoke, physical activity, level of education and medical co-morbidities were not examined. Additionally, based on the tobacco classification definition used, it is possible that persons who used tobacco for limited durations early in adulthood (e.g., ≤10 pack-years) might result in misclassification as former smokers when their risk of incident CVD events is likely much closer to never smokers. Finally, it is also possible that the changes in tobacco use as measured for this analysis were temporary. For example, a sensitivity analysis found that only one-third of those who transitioned to dual use between the first and second waves remained dual users at the third wave, and 16.5% (SE = 2.5) of those who quit between the first and second waves went back to exclusive combustible-tobacco use by the third wave, suggesting the potential for misclassification of tobacco use status. Accordingly, our analytic approach using three wave “windows” likely helped to minimize any misclassification with regard to transient changes in tobacco use status.

## 5. Conclusions

In conclusion, we did not find evidence to suggest that transitioning to exclusive use of ENDS significantly changes the odds of CVD incidence after one year, although this analysis was based on a limited sample size and a relatively short follow-up interval. These findings are in line with other current research showing that among exclusive combustible-tobacco users who became exclusive ENDS users, CVD incidence does not appear to significantly change [27]. It is possible that additional waves of PATH Study data, combined with information from other large longitudinal cohorts with careful tracking of ENDS use patterns, may help to further clarify this relationship.

## Figures and Tables

**Table 1:** Selected demographic and cardiovascular risk factors assessed at baseline, PATH Study adults age 40+ with no cardiac condition at baseline, by tobacco user group.

		Exclusive Combustible-Tobacco Users at Baseline												Never Users of Tobacco			
		Continuing Exclusive Combustible-Tobacco Use			Switch to ENDS (Exclusive Use)			Switch to ENDS (Dual ENDS/Combustible-Tobacco Use)			Quit (no Past 30 Day Tobacco Use)			Remain Past 30 Day Non-Users of Tobacco			
		Obs.	%	SE	Obs.	%	SE	Obs.	%	SE	Obs.	%	SE	Obs.	%	SE	*p*-value
		6263			53			564			654			3014			
Sex	Male	3150	55.3	1.0	24	47.4	7.7	223	44.2	2.2	375	61.7	2.6	935	32.8	1.3	
	Female	3107	44.7	1.0	29	52.6	7.7	341	55.8	2.2	278	38.3	2.6	2067	67.2	1.3	<0.001
Age	40–54	3403	52.9	1.2	30	57.0	7.5	378	66.6	2.0	353	49.4	2.8	1472	45.2	1.7	
	55+	2860	47.1	1.2	23	43.0	7.5	186	33.4	2.0	301	50.6	2.8	1542	54.8	1.7	<0.001
			Mean	SE		Mean	SE		Mean	SE		Mean	SE		Mean	SE	
Average Age			54.6	0.2		52.2	1.6		51.5	0.4		55.7	0.5		57.6	0.5	<0.001
Average Pack-Years			25.1	0.8		16.1	2.9		28.0	2.2		11.2	1.3		N/A		<0.001
Ever Report of:		Obs.	%	SE	Obs.	%	SE	Obs.	%	SE	Obs.	%	SE	Obs.	%	SE	
High Blood Pressure (a)		2510	38.4	1.2	17	32.4	7.3	179	30.2	2.0	252	36.2	2.5	1208	39.4	1.7	0.16
High Cholesterol (a)		1981	32.3	0.9	18	37.2	7.4	166	28.6	2.2	223	38.2	2.5	940	31.1	1.5	0.10
Diabetes (b)		1257	19.6	1.0	13	24.9	6.6	110	18.0	1.9	129	20.6	2.4	723	23.0	1.4	0.08
BMI ≥35 (c)		758	11.3	0.6	8	13.5	5.0 ^	86	13.7	1.5	99	12.4	1.5	495	14.5	0.9	0.01
Family History (d)		377	5.7	0.3	3	4.1	2.5 ^	50	7.7	1.1	29	3.6	0.8	157	5.2	0.6	0.14

Notes: Weighted estimates, unweighted Ns; Baseline, first wave of each interval; Obs., observations; SE, standard error. (a) Has a doctor or other health professional ever told you that you had any of the following conditions? High blood pressure, high cholesterol, congestive heart failure, stroke, and heart attack (no, yes). (b) Has a doctor or other health professional ever told you that you had diabetes? (no, yes). (c) Body mass index (BMI) was calculated for each participant based on their height and weight; elevated BMI was defined as ≥35. (d) Were any of your close biological or blood relatives ever told by a health professional that they had a heart attack or needed bypass surgery? If yes, were they told they had a heart attack or needed bypass surgery before the age of 50? (no, yes). ^ Estimate has been flagged because it is statistically unreliable. It is based on a denominator sample size of less than 50, or the coefficient of variation of the estimate or its complement is larger than 30 percent.

**Table 2:** Cardiovascular disease at follow up by baseline and interim tobacco use status: adjusted GEE results among 9828 observations.

							95% CI		
Baseline Tobacco Use Status	Tobacco Use Status Change	Observations	N with Cardiovascular Disease at Follow Up	%	SE	Adjusted Odds Ratio	Lower	Upper	*p*-Value
Exclusive Combustible-Tobacco Users at Baseline	Continuing Exclusive Combustible-Tobacco Use	6263	126	2.06	0.20	1.44	0.87	2.39	0.15
	Switch to ENDS (exclusive use)	53	0	0.00	0.00				
	Switch to ENDS (dual ENDS/combustible-tobacco use)	564	15	2.47	0.61	1.85	0.78	4.37	0.16
	Quit (no past 30 day tobacco use)	654	10	1.55	0.78	1.18	0.33	4.26	0.80
Never Users of Tobacco	Remain Past 30 Day Non-Users of Tobacco	3014	40	1.47	0.27	Ref			

Notes: Weighted estimates, unweighted Ns; Baseline, first wave of each interval; Obs, observations; CVD, cardiovascular disease; SE, standard error; CI, confidence interval. The GEE model is adjusted for sex, age, cigarette pack-years, ever report of high blood pressure or cholesterol, diabetes, BMI ≥ 35, and family history of premature heart disease.

## Data Availability

Restricted data sets were analyzed in this study and the data is not publicly available. Since restricted use files (RUFs) retain more than minimal risk for reidentification of a research subject, access to RUFs is granted through controlled conditions to vetted researchers and sponsor-supervised students.
